# Succession of Bacterial Community Structure and Diversity in Soil along a Chronosequence of Reclamation and Re-Vegetation on Coal Mine Spoils in China

**DOI:** 10.1371/journal.pone.0115024

**Published:** 2014-12-11

**Authors:** Yuanyuan Li, Hongyu Wen, Longqian Chen, Tingting Yin

**Affiliations:** 1 Jiangsu Key Laboratory of Resources and Environmental Information Engineering, School of Environment Science and Spatial Informatics, China University of Mining and Technology, Xuzhou, China; 2 School of Life Science, Jiangsu Normal University, Xuzhou, China; 3 Faculty of Built Environment, University of Malaya, Kuala Lumpur, Malaysia; University of Milan, Italy

## Abstract

The growing concern about the effectiveness of reclamation strategies has motivated the evaluation of soil properties following reclamation. Recovery of belowground microbial community is important for reclamation success, however, the response of soil bacterial communities to reclamation has not been well understood. In this study, PCR-based 454 pyrosequencing was applied to compare bacterial communities in undisturbed soils with those in reclaimed soils using chronosequences ranging in time following reclamation from 1 to 20 year. Bacteria from the *Proteobacteria*, *Chloroflexi*, *Actinobacteria*, *Acidobacteria*, *Planctomycetes* and *Bacteroidetes* were abundant in all soils, while the composition of predominant phyla differed greatly across all sites. Long-term reclamation strongly affected microbial community structure and diversity. Initial effects of reclamation resulted in significant declines in bacterial diversity indices in younger reclaimed sites (1, 8-year-old) compared to the undisturbed site. However, bacterial diversity indices tended to be higher in older reclaimed sites (15, 20-year-old) as recovery time increased, and were more similar to predisturbance levels nearly 20 years after reclamation. Bacterial communities are highly responsive to soil physicochemical properties (pH, soil organic matter, Total N and P), in terms of both their diversity and community composition. Our results suggest that the response of soil microorganisms to reclamation is likely governed by soil characteristics and, indirectly, by the effects of vegetation restoration. Mixture sowing of gramineae and leguminosae herbage largely promoted soil geochemical conditions and bacterial diversity that recovered to those of undisturbed soil, representing an adequate solution for soil remediation and sustainable utilization for agriculture. These results confirm the positive impacts of reclamation and vegetation restoration on soil microbial diversity and suggest that the most important phase of microbial community recovery occurs between 15 and 20 years after reclamation.

## Introduction

Mining activities result in extensive soil damage, causing drastic disturbances in landscape, altering the ecological environment of soil microorganisms, thereby disrupting the functional stability of the microbial community [1]. The ultimate goal of mine land reclamation is the reestablishment of a productive, healthy and sustainable ecosystem suitable for post-mining land use [2], [3]. Because of the important ecosystem functions mediated by microorganisms in the soil, recovery of the soil microbial community is a critical step in achieving the goal of soil restoration for its sustainable and beneficial use [4]. Currently, criteria for successful restoration have largely been restricted to soil erosion, physicochemical status and vegetation characteristics [5]–[7]. Analysis of microbial ecological indicators such as microbial populations, microbial communities and function diversity in reclamation evaluation were relatively uncommon [8]. Changes in microbial community can precede detectable changes in soil physicochemical properties, thereby providing early signs of environmental stress or ecological environment evolution in the mining area [9]. Although microbial communities regulate important ecosystem processes, it is often unclear how the abundance and composition of microbial communities correlate with reclamation and interact to affect ecosystem processes. Previous studies on the effect of land reclamation have primarily focused on the physical properties, chemical characteristics, or heavy metal pollution of reclaimed mine soil [10]–[12]. There are few studies on general soil microbial community recovery and these studies have revolved around the effects of soil reclamation on microbial populations, microbial biomass and activity [9], [12], [13], [14]. In case of mine soils, recovery of soil bacterial diversity and structure in disturbed and reclaimed lands is not well understood.

Successful reclamation not only depends on the methods of mining, height and slope of dumps, nature of mine soils, geoclimatic conditions but also on the selection of appropriate species and their ameliorative affacts on mine soils [15]. In China, N-fixing species of legumes, grasses and trees are commonly used for reclamation of coal mine degraded land, thus a mix-planting experiment on annual legume with gramineous forage grass was conducted in our study. The establishment of plant cover in degraded land aims to accelerate soil-forming processes, control erosion, build up organic matter, develop microbial communities, initiate nutrient cycling and enhance overall aesthetics of the area [16].

In the present study, the recovery of soil microbial community in reclaimed sites were examined using chronosequences ranging in time following reclamation from 1 to 20 year. The chronosequence approach has effectively been used to examine the effect of time on ecological succession, soil development and vegetation recovery following disturbance [17]–[19]. The chronosequence design can assist in our understanding of recovery of soil microbiota over large time periods, and can provide information critical to manipulate successional processes for restoration [20].

This study was conducted to investigate the response of bacterial communities to land reclamation and re-vegetation of different reclamation ages. The 454 pyrosequencing technology was applied to describe microbial community recovery through time in reclaimed soils and compare reestablished bacterial community structure and diversity in reclaimed mine soils with those found in undisturbed soils. We hypothesized that reestablished microbial diversity and community composition in long-term reclaimed mine soils would be more similar to those in undisturbed soils. We also hypothesized that variations in bacterial communities might also be associated with soil physical-chemical factors such as pH, soil total nitrogen, phosphorus, organic matter and available potassium.

## Materials and Methods

### Ethics statement

In our study, the research samples were reclaimed soils in the coal-mining reclamation land and undisturbed soils in farmland. Permits for access to sampling sites and for collection of soil samples were obtained from the Tongshan Land Resources Bureau. The field studies did not involve endangered or protected species.

### Site description

The study was carried out in the Liuxin reclamation demonstration area in Tongshan District, approximately 10 km north of Xuzhou, northwestern Jiangsu Province, and is dominated by a North Temperate Zone monsoon climate. The area has an average annual temperature of 13.8°C and receives approximately 868.6 mm of annual precipitation, with an average of 486.4 mm occurring as rainfall during the growing season.

An undisturbed reference site (UND), and a series of reclaimed sites (REC) from the Liuxin Coal Mine were chosen for soil sampling, with reclamation treatments including the following recovery times (in years): 1, 8, 15 and 20. All sampling sites were arranged adjacent to one another, allowing for climate, topography, and parent material to be consistent across study sites. REC, a mining reclamation land surface mined during the late-1980s. The REC consisted of four sites including 1-, 8-, 15- and 20-yr-old reclamations comprising of backfilling, topsoil handling, application of organic amendments, planting a grass-legume mixture and mulching with crop residues. Detailed information on the organic manure has been given before [21].The vegetation is dominated by alfalfa (*Medicago sativa*), white clover (*Trifolium repens*) and ryegrass (*Lolium perenne*), which were planted at a sowing ratio of 3∶3∶2. UND, an adjacent (<1 km) natural land that had not been cultivated since the 1980s, was selected as a reference site. At the time of sampling, vegetation in the undisturbed soil was ∼90% arbor and ∼10% wild plants, composed predominantly of arborvitae, elm, locust (*Robinia pseudoacacia L.*), green foxtail (*Setaria viridis*) and eleusine indica.

### Soil sampling and analysis

Soil sampling was conducted in 2013 during periods of active vegetation growth and prior to vegetation senescence. On each reclamation site (REC-1, REC-8, REC-15, REC-20) and one reference site (UND), three 45 m transects were randomly set up, which served as the basis for all sampling. Transects fell beside one another and were separated by 10–20 m from end-to-end. Four randomly located points along each transect served as locations for soil sampling (S1 Figure). Samples of bulk soil were collected at a depth of 0–20 cm from each sampling point by use of an auger (3 cm diameter), resulting in 12 samples per restoration time (four per transect), plus additional 12 samples for the undisturbed soil. Four sampling points were composited and each transect was considered a replicate of each site due to the difficulty in finding reclaimed sites of the same age, and thus we obtained three values for physicochemical parameters and biodiversity indices. When the soil samples were sieved (<2 mm), with aboveground plant materials, roots and stones being removed, all soil were stored on ice upon collection and transported to the laboratory for DNA extraction. A portion of soil samples was air-dried and crushed in a porcelain crucible in order to determine chemical and physical properties (Table 1).

**Table 1:** General physical and chemical properties (n = 3, mean±SE)* of soil for each site.

Site	Site age(yr)	pH	SOM/(g/kg)	Total P/(g/kg)	Total N/(g/kg)	Available K/(mg/kg)
REC	1	8.08±0.06a	7.61±0.81c	0.70±0.17bc	0.81±0.34d	100.54±6.71e
	8	8.07±0.09a	8.79±0.68bc	0.79±0.19c	0.94±0.31c	111.10±4.71d
	15	8.04±0.05a	10.26±0.98b	0.88±0.09b	1.17±0.14b	120.50±2.47c
	20	8.01±0.02a	13.11±1.10a	0.92±0.11a	1.35±0.20a	125.61±4.21b
UND		7.99±0.01a	13.87±1.04a	0.99±0.12a	1.38±0.32a	129. 74±6.21a

n = number of replicates of each site

*Values followed by different letters are significantly different (P<0.05) according to Duncan's multiple comparison.

SOM, Soil organic matter; Total N, Total nitrogen; Total P, Total phosphorus; Available K, Available potassium.

Sites are designated by recovery time (in years)(REC) or as undisturbed reference (UND).

The soil pH was measured with an acidity meter (Sartorius PT-21, Shanghai, China) in a 1∶5 soil-water mixture. Soil organic matter content (SOM) was determined with dichromate oxidation method [22].Total nitrogen (Total N) and phosphorus (Total P) in soil were extracted by Kjeldahl digestion [23] and using ammonium molybdate spectrophotometer [24], respectively. Available potassium (Available K) was determined by ammonium acetate and determined by flame photometry [25].

### DNA extraction, 16S rRNA amplification and pyrosequencing

Genomic DNA was extracted from 500 mg of soil sample using the E.Z.N.A. Soil DNA Kit (Omega, Bio-Tek, Inc., Norcross, GA U.S.) as described by the manufacturer. The genomic DNA concentration and purity were determined by using agarose gel electrophoresis (1%) and microspectrophotometry (NanoDropÒND-2000, Nan Drop Technologies, Wilmington, DE, U.S.). All DNA samples were diluted to equivalent 0.5 ng/µl concentrations for a 20-µl PCR reaction.

The set of primers: 27F (5′-AGAGTTTGATCCTGGCTCAG-3′) and 533R (5′-TT ACCGCGGCT GCTGGCAC-3′) was used to amplify the V2–V3 hypervariable region of the 16S rRNA gene [26]. PCR was carried out in triplicate with 20 µl of the reaction mixture comprising 4 µl of five-fold FastPfu buffer, 2 µl of 2.5 mM dNTPs, 5 µM of each primer, 0.4 µl of diluted DNA sample, 0.4 µl of TransStart FastPfu DNA Polymerase, and approximately 10 ng of DNA template by using the PCR Gene Amp 9700 (Applied Biosystems, Foster City, CA, U.S.).The PCR conditions were 95°C for 2 min, 25 cycles of 95°C for 30 s, 55°C for 30 s, and 72°C for 30 s extension followed by 72°C for 5 min. All samples were amplified in triplicate, pooled in equal amounts, and purified using an Axy Prep DNA gel extraction kit as recommended by the manufacturer (Axygen, Biotechnology, Hangzhou, China). Quantification of the PCR products was performed using the PicoGreen dsDNA Quantitation Reagent (Molecular Probes, Eugene, OR, U.S.) and a Real-time PCR System (Promega, Madison, WI, U.S.) as recommended by the manufacturer. After quantitation, the amplicons from each reaction mixture were pooled in equimolar ratios based on concentration and subjected to emulsion PCR to generate amplicon libraries, as recommended by 454 Life Sciences. PCR products were submitted to Shanghai Majorbio Bio-pharm Technology Co., Ltd. for pyrosequencing using a 454/Roche GS-FLX Titanium Instrument (Roche, NJ, U.S.).

### Analysis of pyrosequencing data

After trimming of the barcodes and primers, bacterial sequences that were shorter than 200 bp in length and reads containing ambiguous bases or any unresolved nucleotides were removed from the pyrosequencing-derived datasets. The trim of the reads was performed by using the bioinformatics software package, seqcln and mother (http://sourceforge.net/projects/seqclean/&http://www. moth ur.org/wiki/Main_Page). After that, reads were taxonomically assigned to a bacterial 16S rRNA Silva reference alignment using a naïve Bayesian classifier. The detection and removal of chimeras were processed using a new program/algorithm, UCHIME [27]. Sequences with similarities of greater than 97% were clustered into one operational taxonomic unit (OTU) using the MOTHUR program [28]. The high-quality sequences were taxonomically classified using the RDP naïve Bayesian rRNA Classifier at confidence level of 80% [29]. For the determination of OTUs, we defined species, genus and phylum level at 3, 5 and 20%, respectively [30]. Community richness and diversity indices (Chao1 estimator, ACE and Shannon index) and rarefaction curves were obtained using the MOTHUR program. The 16S rRNA gene sequences derived from pyrosequencing have been deposited in the NCBI Sequence Read Archive (SRA) under the accession number SRA091276.

### Statistical analyses

The contents of soil organic matter, Total N, Total P, Available K and pH were tested for differences among sites with the one-way Analysis of Variance (ANOVA). Bacterial community richness and diversity indices (ACE, Chao1, Shannon and Simpson) were compared using one-way ANOVA. Pairwise comparisons were made with Dunnett's T3 post-hoc test (at P<0.05) which does not require the assumption of homogeneity of variance and is better suited to handle unbalanced designs. Correlations among physiochemical and microbiological characteristics were conducted by Pearson correlation analysis. Graphing and data analysis were performed with SPSS BASE ver.13.0 statistical software (SPSS, Chicago, IL, U.S.) and OriginLab Origin Pro software (version 9.0) (OriginLab, Northampton, MA, U.S.).

To compare bacterial community structures across all sample sites, principal component analysis (PCA) and hierarchical cluatering analysis were performed using the UPGMA (Unweighted pair group method with arithmetic mean) method [31] and using CANOCO for Windows [32]. Bray–Curtis indices were calculated and represented in a heat map format with hierarchical cluster analysis to depict the similarity and dissimilarity between bacterial communities. The heatmap was made with R version 2.11.0 using the heatmap function [33]. A phylogenetic tree was also constructed from 2842 OTUs based on an evolutionary distance of 0.03 and NCBI GenBank reference sequences using MEGAN [34] (http://ab.inf.uni-tuebingen.de/software/megan/). To examine the relationship between relative abundances of abundant phyla (proteobacterial classes) and soil physicochemical properties, a redundancy analysis (RDA) was carried out [35].

## Results

### Selected soil physicochemical characteristics

To provide physical and geochemical context for microbial community analysis, we compared selected physicochemical parameters of soils from the different aged reclamation sites with those from the undisturbed site. In general, contents of SOM, Total N, P and Available K were consistently lower in the 1-, 8- and 15- yr-old reclamation sites than the undisturbed reference. With an increase in reclamation age, SOM, Total N and Available K generally increased, and the oldest reclaimed site (20-yr-old) had similar levels of these soil variables to the undisturbed reference. On the contrary, soil pH values changed in the range of 7.99-8.08 across all sites, and slightly declined with reclamation time increased.

As is shown in Table 1, the contents of SOM tended to be lowest in the most recently reclaimed site (1-yr-old), significantly lower than the 15- and 20-yr-old reclaimed sites as well as the undisturbed site, indicating a reduction in the variability of observed SOM values following disturbance at the initial stage. But as recovery time increased, SOM gradually increased, with higher SOM values in the 20-yr-old site similar to the undisturbed site. REC-20 contained about 72.27% higher SOM than REC-1, indicating that SOM increased markedly as reclamation time increased. The contents of Total N and Available K also significantly increased (P<0.05, Table 1) in older reclaimed sites after 15 or 20 years of reclamation relative to the youngest reclaimed site, with an increase of 60.49% and 24.93%, respectively. Overall, similar amount of SOM, Total N, P and Available K were present in UND and REC-20, both of which were significantly greater than REC-1, suggesting that long-term reclamation practice and re-vegetation greatly improved physicochemical properties of degenerated soil to nearly pre-disturbance levels.

### Bacterial community diversity

Nonparametric indicators (Chao1, ACE, Shannon and Simpson) were used to present community diversity of bacteria associated with reclaimed soils of different ages and the undisturbed soil. This comparison of richness and diversity indices revealed differences in the complexity of the bacterial communities of these studied soil sites, suggesting a great reduction of bacterial diversity level following reclamation especially within the first few years of recovery. However, richness and diversity indices tended to increase with increasing time of reclamation.

As shown in Table 2, the most recently reclaimed soil was found to have significantly lower diversity indices compared to the undisturbed soil. REC-1 had the lowest richness indices for Chao1 (1200±10) and ACE (1287±18) and the lowest diversity for Shannon (5.17±0.02). More complex microbial communities tended to be associated with older reclaimed sites (REC-15 and REC-20) and UND in comparison to younger reclaimed sites (REC-1 and REC-8). After 20-yr reclamation, the reclaimed site had similarly higher mean values for species richness indices of Chao1 (5426±302), ACE (7892±650), and diversity indices of Shannon (7.06±0.06) than the undisturbed soil, but the differences were not significant. The following trend was found in terms of Simpson indices at 3% among sites: UND<REC-20<REC-15<REC-8<REC-1, which confirmed much higher diversity levels of bacterial communities belonging to older reclaimed soils than younger reclaimed soils.

**Table 2:** Estimated number of observed OTUs (at 97% similarity), richness, diversity, and coverage (n = 3, mean±SE)* of soil for each site.

Site	Site age (yr)	OTU_97%_	Coverage	Richness and diversity indices*			
				Chao 1	ACE	Shannon	Simpson
REC	1	1110±30b	0.89±0.01a	1200±10c	1287±18c	5.17±0.02c	0.0233±0.00a
	8	1327±32b	0.89±0.03a	1398±30c	1446±30c	5.89±0.01b	0.0119±0.00b
	15	1406±45b	0.91±0.22a	2458±83b	3569±256 b	5.79±0.02b	0.0085±0.00c
	20	2755±100a	0.93±0.07a	5426±302a	7892±650a	7.06±0.06a	0.0028±0.00d
UND		2243±105a	0.88±0.01a	4670±260a	5699±505ab	7.11±0.06a	0.0013±0.00d

n = number of replicates of each site

*Different lowercase letters indicate statistically significant differences (P<0.05) between soil sites according to Duncan's test at P<0.05.

OTUs, operational taxonomic units; Coverage, Good's nonparametric coverage estimator; ACE, abundance-based coverage estimator; Shannon, nonparametric Shannon diversity index; Simpson, nonparametric Simpson diversity index.

Sites are designated by recovery time (years) or as undisturbed reference (UND).

**Figure 1:**
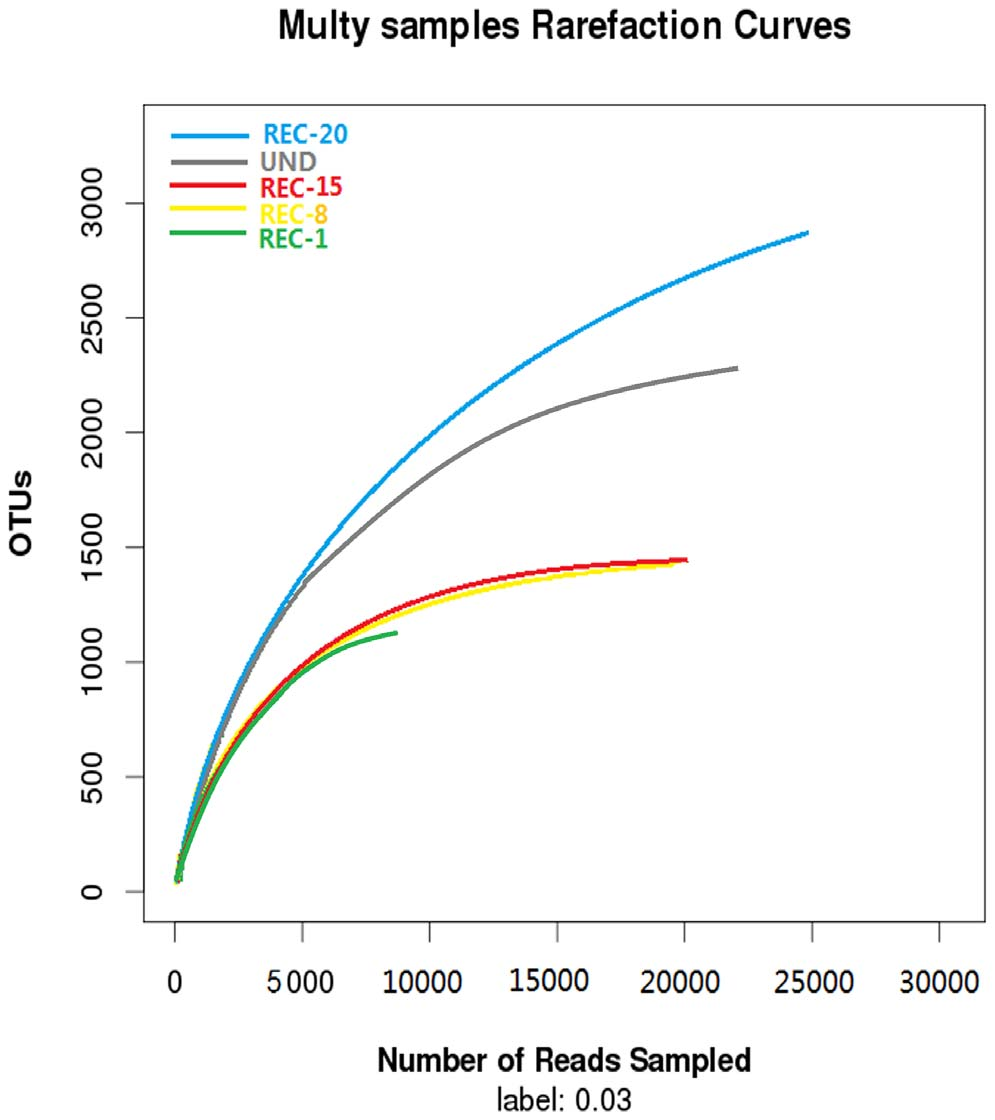
Rarefaction analyses of samples. Rarefaction curves of OTUs clustered at 97% sequence identity across different samples from reclamation sites of different ages (REC) and the undisturbed reference (UND). UND refers to site undisturbed, REC-1 to site reclaimed for 1 year, REC-8 to site reclaimed for 8 years, REC-15 to site reclaimed for 15years and REC-20 to site reclaimed for 20 years.

**Figure 2:**
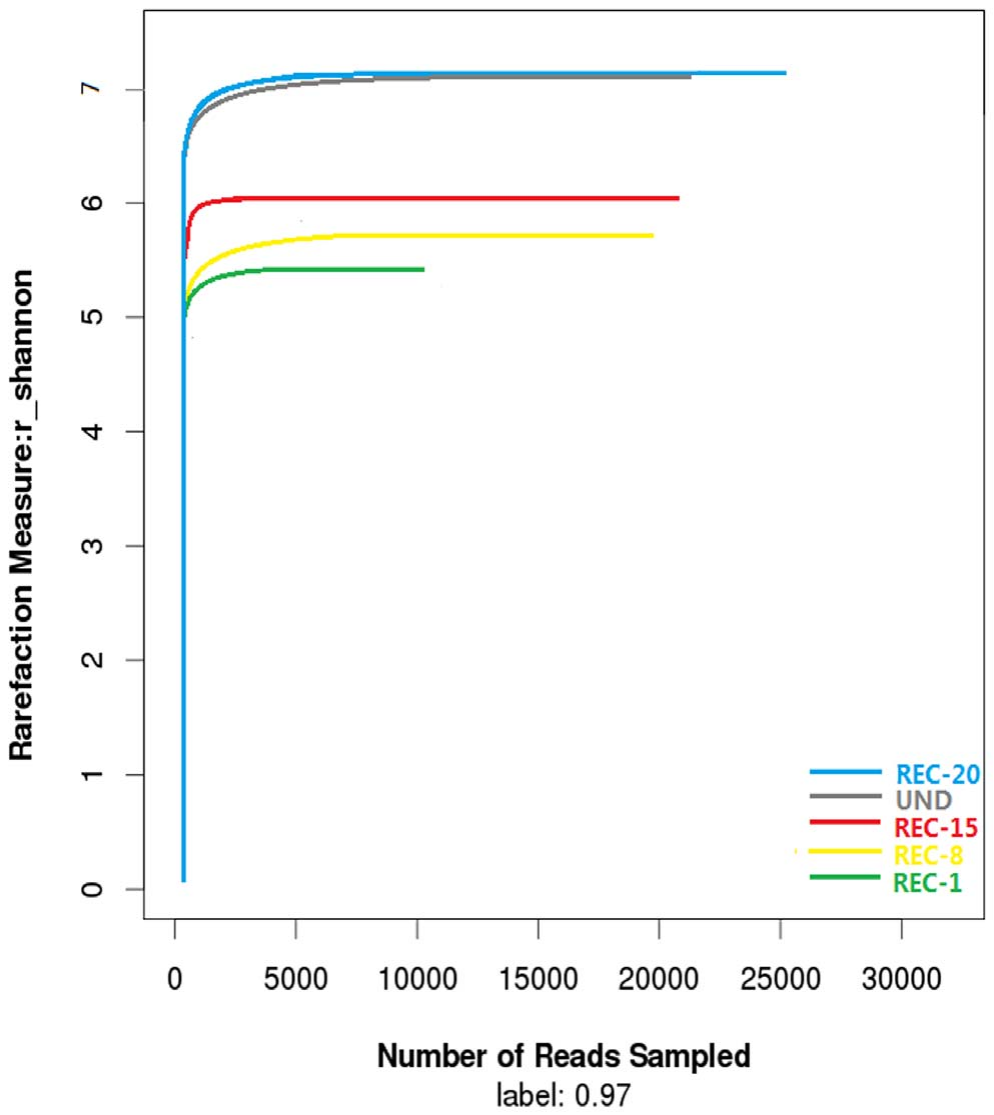
Shannon Wiener curves of samples. Shannon Wiener curves of OTUs clustered at 97% sequence identity across different samples from reclamation sites of different ages (REC) and the undisturbed reference (UND).

The comparison of rarefaction curve and Shannon Wiener curves showed similar trends (Fig. 1 and Fig. 2), and the following trend was found in terms of high species richness at 3% dissimilarity: REC-20>UND>REC-15>REC-8>REC-1. The older reclaimed sites exhibited relatively high species abundance and diversity as compared to the younger ones, with highest number of observed OTUs for REC-20 and lowest for REC-1. As is shown in Fig. 1 and S2 Figure, the maximum OTU value for REC-20 and UND was ∼3000 and>2000, respectively, while the REC-1 OTU value was <1500. The shapes of Shannon Wiener curves (Fig. 2) also confirmed that bacterial communities in older reclaimed soils are more diverse than the younger reclaimed ones.

### Bacterial community composition

**Figure 3:**
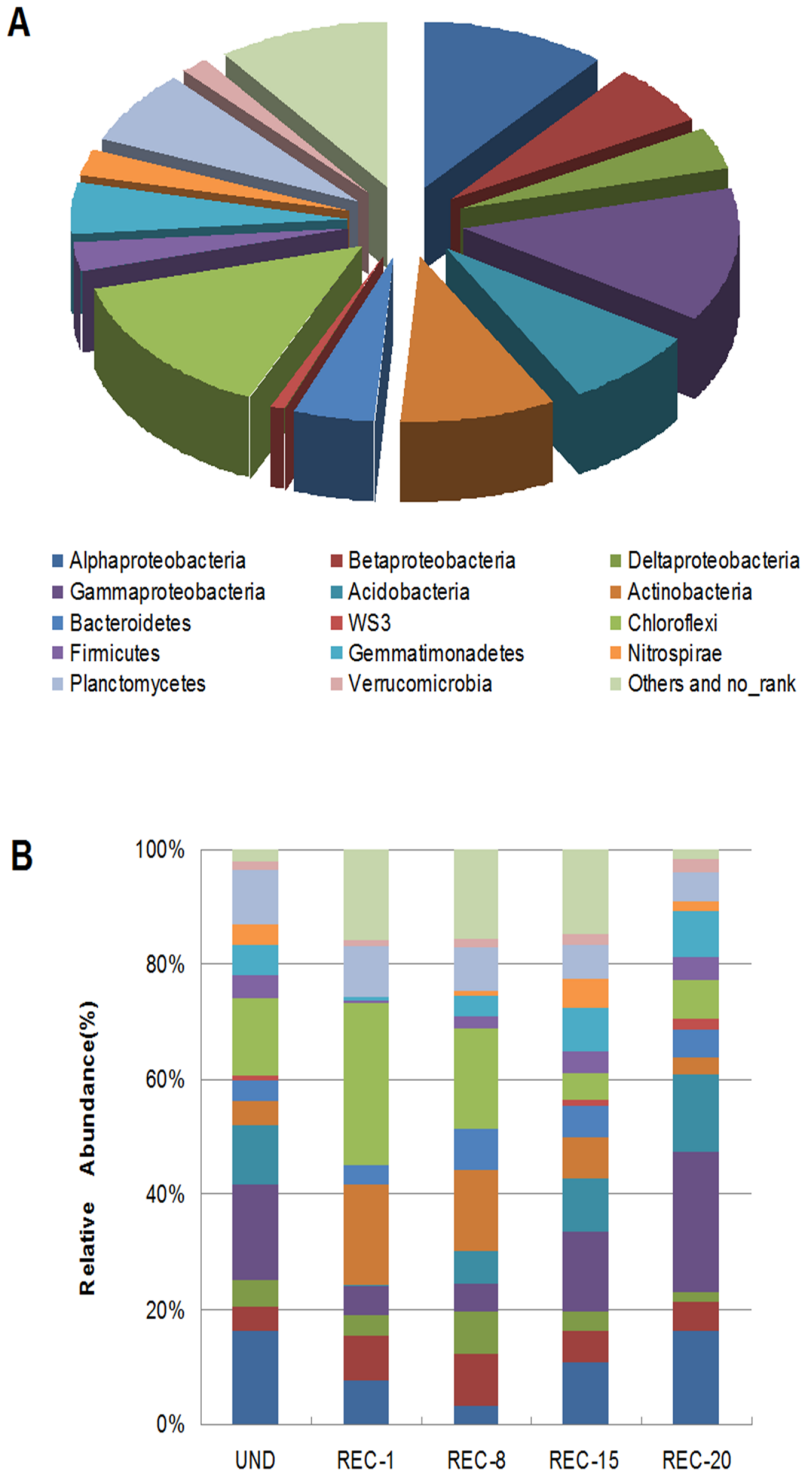
Relative abundance of the dominant bacteria phyla in all sites combined (A) and in each site (B). Relative abundances (>1%) are based on the proportional frequencies of those DNA sequences that could be classified at the phylum (proteobacterial class) level. Phylogenetic groups accounting for ≤1% of all classified sequences are summarized in the artificial group “Others”.

In order to analyze soil bacterial community composition, we applied 454 pyrosequencing of the V2-V3 region of the 16S rRNA gene. Sequences that could not be identified were removed from the pyrosequencing-derived dataset and excluded from subsequent analyses. A total of 98,133 high-quality sequences were obtained comprising all samples, with an average length of 480 bp. 9,936, 20,087, 20,675, 24,541 and 22,894 sequences were obtained from samples of REC-1, REC-8, REC-15, REC-20 and UND, respectively. Sequences belonging to 32 different bacterial phyla, 81 classes, 133 orders, 195 families, 286 genera, and 299 species were identified across all sites. Phylogenetic analysis indicated that predominant phyla in all soils were *Acidobacteria* (7.82%), *Actinobacteria* (9.20%), *Bacteroidetes* (4.80%), *Chloroflexi* (14.11%), *Gemmatimonadetes* (4.97%), *Planctomycetes* (7.31%) and *Proteobacteria* (34.11%), accounting for 82.32% of the bacterial sequences (Fig. 3A and S1 Table). In addition, *Firmicutes* (2.90%), *Nitrospirae* (2.28%), *Verrucomicrobia* (1.64%) and WS3 (0.97%) were present in all of the samples with low abundance.

Soil bacterial community compositions and their differences among all sites were clearly distinguished by pyrosequencing. Different patterns of community structure were observed at the phylum and genus levels across all sites (Fig. 3B and S3 Figure). Changes in soil bacterial community structures were also observed among reclaimed sites of different ages, showing some variation of abundance of each group by the timing of reclamation. *Acidobacteria* made up 10.25%, 13.51% and 9.58% of the total bacterial communities at UND, REC-20 and REC-15, but were present in lower percentages at REC-1(0.2%) and REC-5(5.58%). Likewise, relative abundances of *Proteobacteria* -affiliated phylotypes tended to be more abundant in older reclaimed sites (REC-20: 47.23%; REC-15: 33.20%), and slightly abundant in younger (REC-1: 23.94%; REC-15: 24.57%) and undisturbed site (21.62%). Relative abundances of *Actinobacteria* -affiliated phylotypes showed the different pattern, being more abundant in the younger reclaimed sites (REC-1: 17.52%; REC-8: 14.01%) than in older ones (REC-15: 7.08%; REC-20: 2.99%), showing some variation of abundance of each group by the timing of reclamation. Similarly, *Chloroflexi* was more prevalent in newly rehabilitated sites, accounting for 28.2% (REC-1) and 17.59% (REC-8) of the total bacterial communities, respectively. However, it only accounted for 4.71% (REC-15) and 6.60% (REC-20) in older sites. Classes *Alphaproteobacteria*, *Betaproteobacteria*, *Gammaproteobacteria* and *Deltaproteobacteria*, all part of the most dominant phylum *Proteobacteria*, had a markedly different abundance among different sampling sites as well: *Alphaproteobacteria* and *Gammaproteobacteria* groups were more abundant in older reclaimed sites than younger ones, while the *Betaproteobacteria* group was abundant in younger sites. It is noted that *Nitrospirae* and WS3 appeared distinctively in UND and REC-20 while being negligible or absent in younger sites (REC-1 and REC-8).

### Bacterial community structure and phylogenetic analysis

**Figure 4:**
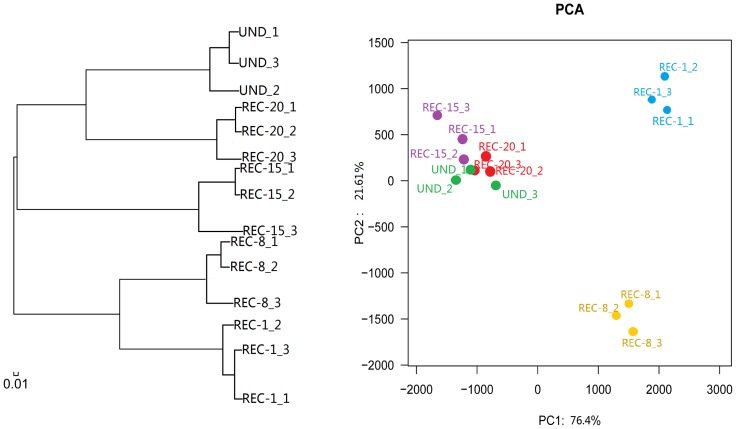
Cluster tree (left) and PCA (right) analyses for soil bacterial communities from reclaimed sites (REC-1, REC-8, REC-15, REC-20) and undisturbed site (UND). The hierarchical clustering analysis of bacterial communities based on Bray–Curtis distance calculated by OTUs at a distance of 3% for soil samples. The scale bar indicates an estimated change of 1%. Principal component analyses (PCA) of soil bacterial communities based on OTUs at a distance of 3%.

To compare the similarity and dissimilarity between all sample sites, hierarchical cluatering analysis and principal component analysis (PCA) were performed (Fig. 4A and S4 Figure). The hierarchical clustering of libraries revealed three main groups (Fig. 4A), the first consisting entirely of samples from younger reclaimed sites (REC-1 and REC-8), the second consisting primarily of samples from the older reclaimed site (REC-15) and the third consisting mainly of libraries with oldest reclaimed (REC-20) and undisturbed site (UND). Hcluster_tree showed that the bacterial communities obtained from the soils reclaimed for 20 years were similar to undisturbed soils, and the samples in 1-yr-old reclaimed soils were similar to 8-yr-old reclaimed soils. Cluster analysis on this data revealed that the undisturbed site was more similar to the 20-, 15-yr-old reclaimed sites, but distinctly different than the 1-, 8-yr-old reclaimed sites.

Concomitantly, principal component analysis (PCA) clearly showed bacterial community variations among these reclaimed soil sites of different restoration time (Fig. 4B), showing some variation of abundance of each group by the timing of reclamation. The first and second principal components explained 76.4% and 21.61% of the variance, respectively. The bacterial communities in the soils from sites REC-15, REC-20 and UND formed a cluster in quadrant II; samples from REC-1 and REC-8 belong to the other quadrants. As seen from the PCA (Fig. 4B), the profiles of bacterial communities in REC-15, REC-20 and UND were separated from those in REC-1 and REC-8 along PC 1, and PC 2 separated bacterial communities in REC-8 from those in REC-1, REC-15, REC-20 and UND. Profiles of soil bacterial communities from 15-, 20- yr-old reclaimed sites and undisturbed site tended to group together, and were clearly separated from 1- and 8-yr-old reclaimed sites. Two younger reclaimed sites (REC-1 and REC-8) were far away from the origin, indicating that land reclamation greatly influenced soil bacterial community especially for the soil reclaimed for only 1 and 8 years.

**Figure 5:**
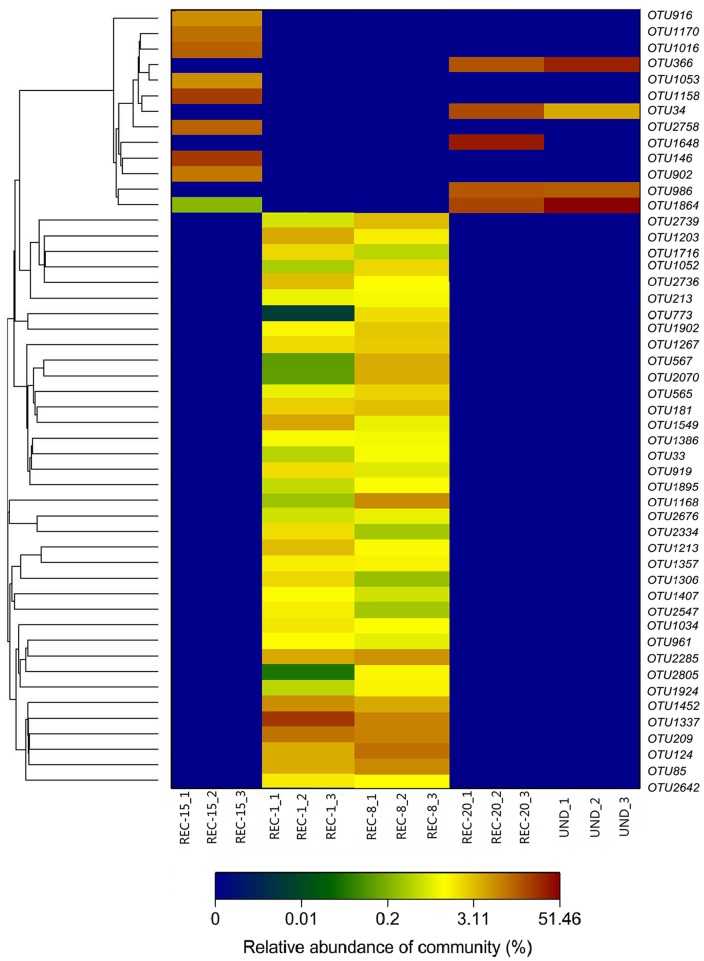
Heatmap and accompanying cluster analyses (x-axis) of all samples based on Bray–Curtis distance indices calculated by OTUs at a distance of 3%. Percentages below the map indicate the abundance of each OTU relative to all bacterial sequences in soils that were classified in each of the 5 sites. The relative abundance for each OTU in different sites is colored in shades of blue (low relative abundance) to green, yellow or brown (high relative abundance) as shown in the color key (bottom).

**Figure 6:**
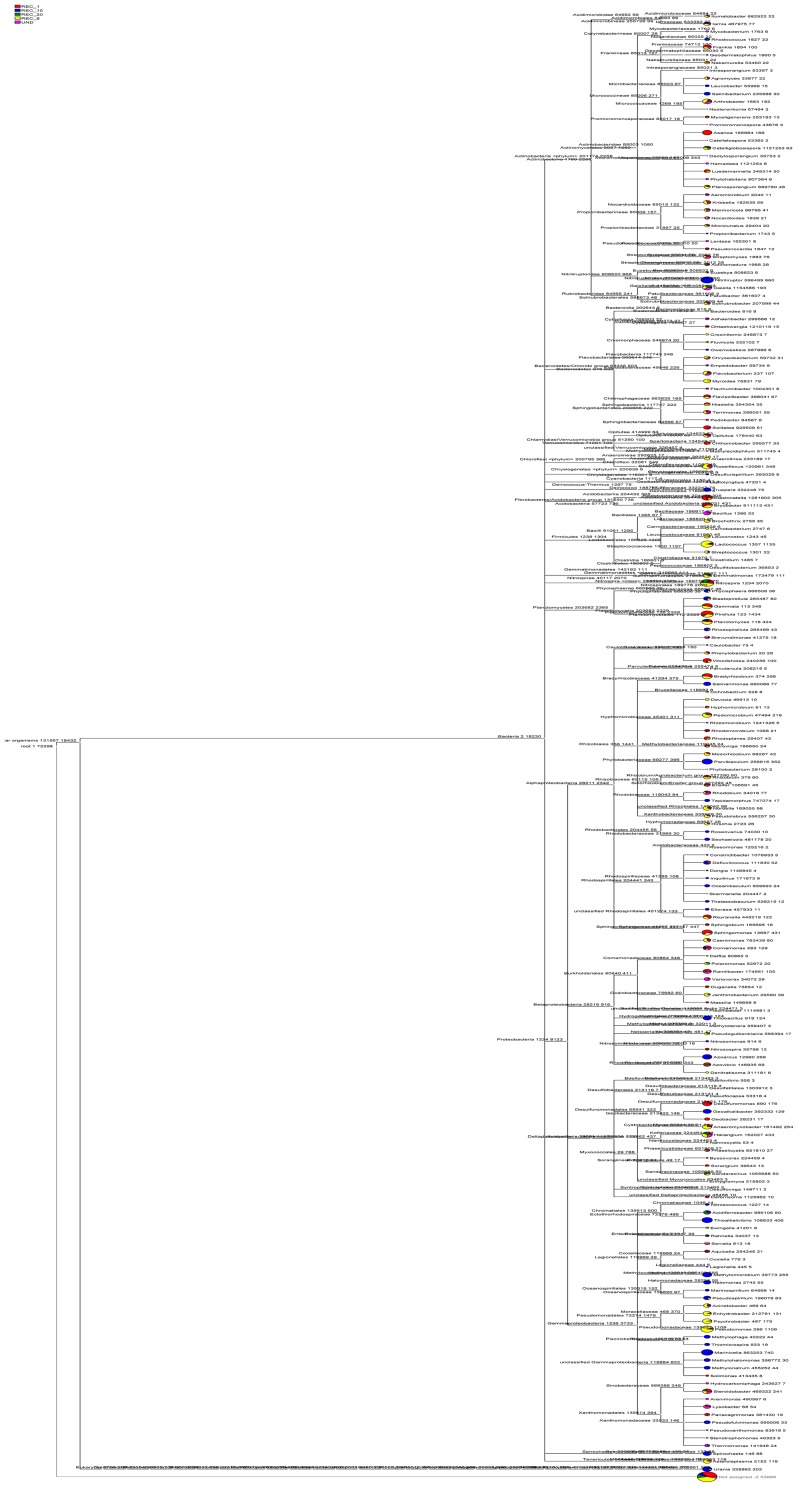
MEGAN integrative taxonomic analysis of bacterial 16S rRNA data sets in multi samples form REC-1, REC-8, REC-15, REC-20 and UND. Taxonomy analysis tree of all sample sites from 2842 OTUs based on an evolutionary distance of 0.03 (species level) and NCBI GenBank reference sequences.

We confirmed this observation by complete hierarchical clustering and heatmap analysis (Fig. 5). The hierarchical heatmap of microbial community was generated with hierarchical cluster based on Bray–Curtis distance indices, displaying the relative abundances of bacterial communities across a wide range of soil samples (UND, REC-1, REC-8, REC-15 and REC-20). The scale bar represents percent abundances within each OTU and values ranged from 0 to 51.46% abundance. Moreover, the color in heatmap also displayed the prevalent group in different samples. Comparison of the relative abundances revealed significant differences between younger (REC-1 and REC-8) and older reclaimed (REC-15 and REC-20) soil bacterial communities. OTU1337, 209, 124, 1452 and 2285 were present in higher proportions in REC-1 and REC-8 compared to REC-15 and REC-20 (P<0.05). Fig. 5 implies that the REC-1 was more similar to the REC-8 samples. Both of the two sites also differed greatly from the undisturbed site. Soil bacterial community structure changed significantly with increasing number of years since reclamation, and were more similar to predisturbance levels(undisturbed) nearly 15 or 20 years of recovery. Relative abundances of soil bacterial community in REC-20 were the most similar to those in UND. For example, OTU34, 366, 986 and 1864 showed a higher relative abundance in soils from REC-20 and UND, while showed a lower relative abundance in soils from REC-1 and REC-8. Moreover, a phylogenetic tree was also constructed for multi samples from REC-1, REC-8, REC-15, REC-20 and UND sites based on an evolutionary distance of 0.03 and NCBI reference sequences (Fig. 6). The color of each site in small pie charts displayed the relative abundance of bacterial community group in different samples sites.

All these analyses suggested that the dominant bacterial phyla observed in our older reclaimed soils (REC-20 and REC-15) and control soils were present in similar relative abundances, and the 20-yr-old reclaimed soil were more similar to the undisturbed soil than other soils.

### Effect of physicochemical factors on soil bacterial diversity and community composition

To address the relationship between the microbial diversity parameters and soil properties, Pearson correlation analyses were built for each variable soil parameters, species richness (Chao1, ACE) and diversity indices (Shannon and Simpson). Correlation analysis indicated that, in undisturbed and all reclaimed soils, SOM showed significant positive correlations with Chao1, ACE and Shannon (r = 0.571-0.729, P<0.01; P<0.05) as show by Table 3, and significant negative correlations with Simpson index (P<0.05). Similar correlations were obtained between soil Total N and these bacterial diversity parameters (Chao 1, ACE, Shannon and Simpson). Bacterial diversity parameters were also in significant correlation with the content of pH and Total P (P<0.05), but were not significantly related to Available K.

**Table 3:** Correlations among soil properties and microbiological diversity parameters.

	Chao 1	ACE	Shannon index	Simpson index	SOM/(g/kg)	TN/(g/kg)	TP/(g/kg)	AK/(mg/kg)	pH
SOM/(g/kg)	0.771**	0.801**	0.867**	-0.813*	1				
TN/(g/kg)	0.789**	0.821**	0.847**	-0.807*	-	1			
TP/(g/kg)	0.715*	0.729*	0.792*	-0.659	-	-	1		
AK/(mg/kg)	0.791	0.778	0.895	-0.889	-	-	-	1	
pH	0.716*	0.688*	0.847*	-0.806*	-	-	-	-	1

**Significant at *P*<0.01; *significant at *P*<0.05; insignificant correlations were omitted

SOM, Soil organic matter; Total N, Total nitrogen; Total P, Total phosphorus; Available K, Available potassium

**Figure 7:**
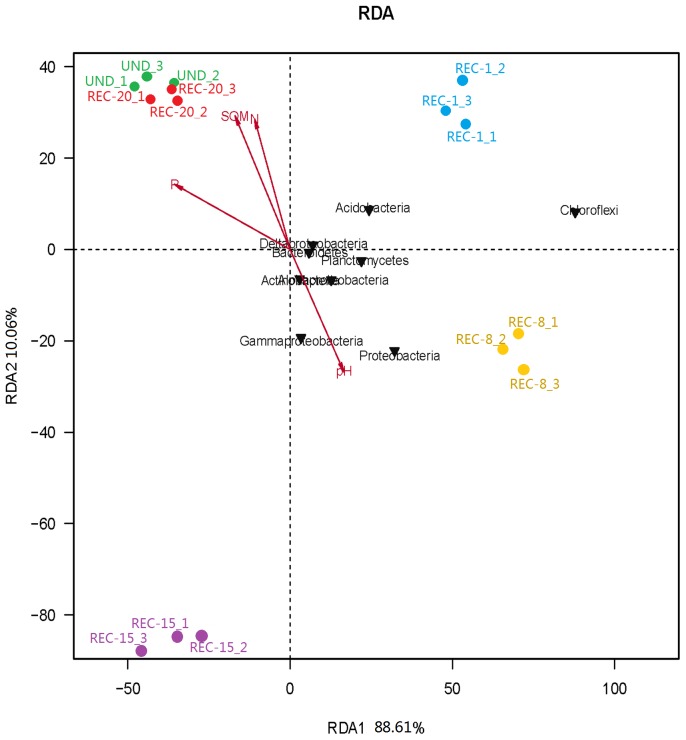
Redundancy analysis (RDA) of abundant phyla (proteobacterial classes) and selected soil edaphic properties such as SOM, total N, total P, and pH for individual samples from undisturbed and reclaimed sites along a chronosequence of reclamation.

Redundancy analysis (RDA) was performed to explore the effect of soil properties on abundant phyla (proteobacterial classes) (Fig. 7). The first two axes of RDA explain 88.61% and 10.06%, respectively, of the total variation in the data. The first axis was related to variation in bacterial populations associated with younger reclaimed sites (REC-1 and REC-8) (plotted on the far positive side of axis 1) and the older reclaimed site (REC-15 and REC-20) (plotted nearer the origin and on the negative end of axis 1). Abundant phyla of the UND and REC-20 were more alike and related to higher SOM, Total N and P contents, as shown by their close grouping and by the vectors. On the other hand, bacterial communities of REC-8 associated with higher pH values. SOM, Total N and P relatively grouped together with the same directionality (Fig. 7) and showed negative correlations with axis 1, while pH showed the positive correlation. Among those selected soil factors, soil Total P was the most important variables associated with the first axi. We found that dominate bacterial phyla (proteobacterial classes) such as *Actinobacteria*, *Alphaproteobacteria*, *Gammaproteobacteria*, *Planctomycetes* and *Proteobacteria* were spread in quadrant I and IV. The relative abundance of those abundant phyla was positively correlated with pH and negatively correlated with SOM, total P and N. We also found that *Acidobacteria* and *Chloroflexi* had no significant correlation with all soil properties.

The second axis was correlated with soil total N and pH. *Gammaproteobacteria* and *Proteobacteria* were associated with relatively high soil pH values, while *Bacteroidetes* and *Deltaproteobacteria* were associated with relatively low soil pH. However, *Gammaproteobacteria* and *Proteobacteria* were associated with relatively low soil total N.

## Discussion

Through cultivation-independent molecular analyses, we provide an assessment of response and recovery of soil microbial communities to disturbance caused by mining reclamation. In the present work, we were able to classify 98,657(94.23%) of the 104,698 quality sequences below the domain level. The total number of analyzed sequences and the percentage of classified 16S rRNA gene sequences exceeded those of other pyrosequencing-based studies of soil bacterial community composition in restoration and mining sites [36]-[37]. Furthermore, at 3% distance (species level), the OTUs identified (ranged from 1136 to 2997) were greater than those reported in other 16S rRNA clone library-based studies [37]-[39].

Results show that the soil bacteria were mainly composed of *Acidobacteria*, *Actinobacteria*, *Bacteroidetes*, *Chloroflexi*, *Gemmatimonadetes Planctomycetes* and *Proteobacteria*(Fig. 3A). This phylum-level profile is similar to other soil and environments [38], [40], [41]. Overall, along with timing of reclamation, bacterial community shifts across these reclaimed soils followed specific trends, such that (a) an increase in *Acidobacteria*, *Proteobacteria*, *Firmicutes* and *Gemmatimonadetes* was shown in older rehabilitated sites, (b) a decrease of *Actinobacteria* and *Chloroflexi* was shown in older rehabilitated sites, and (c) no consistent trend was shown across reclamation sites(*Bacteroidetes* and *Planctomycetes*) (Fig. 3B).Regardless of reclamation ages, *Proteobacteria* was the most abundant, and this finding was generally consistent with those of some researchers who demonstrated that *Proteobacteria* was the most ubiquitous and common group in soil [37], [42]-[44]. Thus, despite sites undergoing different ages of reclamation and surveying efforts in the different studies, *Proteobacteria* was the most advantageous bacteria in a variety of soils.Similar to our study, an increasing number of *Proteobacteria* and *Acidobacteria* with increasing restoration ages has also been recently reported by Banning *et al*. [45]and Lewis *et al*. [46] in post-mined Chronosequence soils from Jamaica and Australia undergoing restoration for 18 and 20 years after cessation of bauxite mining. This suggests that despite decades of rehabilitation, nutrient limitation, among other factors, bacteria belonging to *Proteobacteria* and *Acidobacteria* played a strong likelihood of functional role in soil restoration processes. We found γ-*Proteobacteria* was the largest sub-group of *Proteobacteria* in reclaimed sites, and the populations of specific denitrifying bacteria-*Pseudomonas* or *Pesudomonadales* below the γ-subclass of *Proteobacteria* were observed in significant proportions in those samples. This may indicate a significant role for this nitrogen-cycling bacterium functional group in this mine ecological system because *Pseudomonas* is key to nitrogen cycling of soil [47], [48]. Furthermore, given the fact that *Pseudomonas*-related phylotypes have heavy metal detoxification mechanisms for arsenic, ferrum, and manganese, finding those functional groups in the coal-mining area may have ecological significance because of their important roles in purifying contaminated mine soil [49].

In the current study, we observed a significant alteration in the microbial community structure following land reclamation, with considerably lower abundance and diversity of bacterial community in most recently reclaimed soils comparable to undisturbed soils. Long-term reclamation greatly affected microbial community structure and diversity, which is in agreement with previous studies investigating the shifts in soil-associated microbial communities that occur upon completion of mining activities or reclamation [45], [46], [50]. Similarly, the reduction in microbial communities in response to disturbance and reclamation has been previously documented [5], [12], [51], but has also been observed to require a longer time period (i.e. 20 years) to reach undisturbed levels [9], [52]. Research on the recovery of soil microbial communities through time in surface mines is very rare and not well understood [5], [9]. Our results showed that bacterial communities from the undisturbed site had most abundant species among all sites and its structure and component were very complicate, including the maximum number of phyla (27). The younger reclaimed sites showed relatively simple diversity, and the REC-1 contained the lowest number of phyla (19) among all sites. However, compositions for all soil bacterial communities changed over time, with distributions of bacterial communities generally becoming more similar to those of the undisturbed site as recovery time increased. Principal components analysis (PCA) based on the relative abundances of the different bacterial phyla and proteobacterial classes confirmed that bacterial community compositions shift greatly among all sites along the chronosequence (Fig. 4B). The bacterial communities in control (UND) and older reclaimed soils (REC-15 and REC-20) differed greatly from communities in most recently reclaimed soils (REC-1 and REC-8). PCA and cluster analysis indicated that bacterial communities in undisturbed and 20-yr-old reclaimed soils were more similar to each other than either was to those from the sites rehabilitated for 1, 8 and 15 years. The oldest reclaimed soil and undisturbed soil seemed to harbor more similar bacterial assemblages, indicating that the microbial community structure appeared to recover with time. Consistent with the results we report here, a number of other studies have observed the microbial community in reclaimed soils of varying ages (1-26 yr) to be generally comparable to that found in undisturbed soils, suggesting that recovery has occurred [9].

The results obtained by other studies indicate that coal mining disturbance and reclamation caused the degradation of soil fertility, resulting in the decrease of species diversity, negatively affecting the structure, function and stability of soil microflora, especially during the early reclamation period [5], [9], [18], [53]. In our study, the values of species richness and diversity tended to increase with age of reclamation but in the short term (within 1–8 yrs), remained largely reduced relative to the undisturbed site. Reclamation activities involving soil stripping, reapplication, and tillage are known to have detrimental impacts on soil system, including loss of vegetation, destruction of soil structure, an initial increase in soil organic matter mineralization, compaction, dilution of nutrients, and loss of soil biota. After the reclamation, the restructured soil were in a inhomogeneous mixture, containing parts of deep cultivated soil, large parts of unmatured soil without tillage, as well as inorganic constituent. The consequences of physical disturbance to the top soil during stripping, stockpiling, and reinstatement cause unusually large N transformations and movements with eventually substantial loss. Besides, as filling materials for reclamation, a lot of coal ash and gangue interfere with the energy flow, material cycle and the water-heat conditions in soil. All these factors altered the ecological environment of soil microorganisms, and thereby causing significant reductions of bacteria communities in soil.

The interaction between soil and microorganisms is the driver of ecosystem functions and any modification of this relationship might affect the microbial structure, which, in turn, will influence the ecological processes [54]. In our experiment, Pearson correlation analysis and RDA analysis revealed the effect of physicochemical factors on soil bacterial diversity and community composition, which suggested that the variation in bacterial community abundance and diversity could be partially attributable to changes in the soil properties. Pearson correlation analysis showed that ACE, Chao1 and Shannon indices showed positive relationships with the concentrations of SOM, Total N and P(P<0.01 or P<0.05). Thus, the observed reductions in the contents of SOM, Total N and P following reclamation within 1–8 yrs could explain the declining in the diversity of microbial community during the early reclamation period. Generally, soil fertility tended to modify with increasing time of reclamation, with accumulation of SOM and nutrient elements [55], [56], and approached a relatively stable level nearly 20 yrs after reclamation. This conclusion is consistent with recently studies conducted by other researches [10], [11], [55]. Physicochemical properties of reclaimed soils significantly improved over time, resulting in elevated bacterial diversity/richness indices and making for restoration of microbial diversity that approximated pre-disturbance levels [14], [54]. RDA analysis examined (Fig. 7) the relationship between relative abundances of abundant bacteria phyla and selected soil factors, clearly explaining the main explicable variation of the community exerted by reclamation. The results of RDA showed that relative abundances of most abundant phyla (*Actinobacteria*, *Alphaproteobacteria*, *Gammaproteobacteria*, *Planctomycetes* and *Proteobacteria*) were significantly correlated with pH, SOM, total P and total N (Fig. 7). Soil pH was a significant environmental factor affecting microbial communities except for *Acidobacteria* and *Chloroflexi*. Overall, UND and REC-20 showed the characteristics of more mature soil development as measured by relatively higher SOM, TN and TP than those in the other reclaimed sites. Overall, these data stress the significant role of soil nutrients in shaping bacterial community abundance and diversity. Ecosystem recovery following disturbance requires a longer time period of the reestablishment of key soil biogeochemical processes.

Fundamentally, long-term re-vegetation exerted a significant influence on soil physicochemical properties, which in turn affected bacterial richness and abundance. Re-vegetation constitutes the most widely accepted and useful way to reduce erosion and protect soils against degradation during reclamation [57]. Re-vegetation facilitates the development of N-fixing bacteria and mycorrhizal association, which are fundamental for maintaining the soil quality by mediating the processes of organic matter turnover and nutrient cycling [58]. Reclaimed soils examined in our study are characterized by higher contents of SOM, Total N, Total P, and increased bacterial diversity indices, confirming that years of native vegetation management led to significant beneficial effects on soil physical-chemical properties and bacterial community functions. Coal mine degraded lands are reclaimed by planting drought resistant, fast growing plants without considering their ameliorative properties. The mixture mode of gramineae herbage and leguminosae herbage was constructed because of their adaptation to deficiency of nutrients and fast growing traits. *Medicago sativa* and *Trifolium repens* have been widely planted in East China Plain due to their strong tolerance to drought and barren soil, as well as outstanding properties of heat and cold resistance [59]. Also, nitrogen fixing species (legumes) have a dramatic effect on soil fertility through production of readily decomposable nutrient rich litter and turnover of fine roots and nodules. The community coexistence mechanisms of deep legume roots and shallow grass roots can give full play to the ability of nitrogen fixation and transportation in legumes, and ensure adequate mineral element in grass [60]. It has also been found that the organic substances supplied by the plant roots may stabilize soil aggregates directly or indirectly by providing a source of energy for microorganisms [61].Vegetation growth in reclaimed soils by planting legume with gramineae shall be able to restore the soil fertility and accelerate ecological succession, which in turn promote a more abundant microbial community. The higher bacterial diversity of reclaimed soils highlights an ecological reaction of plant-cover development in reclamation sites, which with consequent litter and root exudate production may create conditions conducive to the eco-diversity and stability of microbial community. These findings suggest that the development of soil microbial communities in relation to the recovery vegetation and vegetation choice, and may therefore represent an important aspect of the ecological restoration of mine degraded soil.

## Conclusion

Reclamation and Re-vegetation produced a significant effect on soil bacterial diversity and community structures. In accordance with our initial hypothesis that bacterial community in older reclaimed soils would be more similar to those in natural soils, we found that bacterial richness and diversity increased significantly with increasing number of years since reclamation, recovering to pre-disturbance levels nearly 20 yrs after reclamation. There was a clear association between bacterial community and soil physicochemical properties such as SOM, TP, TN and pH. The increase of soil variables in reclaimed treatments can be attributed to the mixed-planting experiments on legumes with gramineous grass. We concluded that soil remediation and re-vegetation has indirect effects on soil microbial community diversity through their influence on soil physicochemical properties, especially nutrient elements. Our results also indicated that restoring the variability of soil physicochemical and microbial diversity level similar to the undisturbed soils requires a longer reclamation history.

## Supporting Information

S1 Figure **The schematic sampling map.** UND refers to site undisturbed, REC-1 to site reclaimed for 1 year, REC-8 to site reclaimed for 8 years, REC-15 to site reclaimed for 15years and REC-20 to site reclaimed for 20 years.
(TIF)

S2 Figure **Rarefaction curves indicating the observed number of OTUs at a genetic distance of 3%.** A, B and C represent three replicates for each site, respectively. Sites are designated by reclamation time (in years) (REC) or as undisturbed reference (UND).
(ZIP)

S3 Figure **Microbial community barplot with cluster tree at the genus level.** Phylogenetic groups accounting for ≤1% of all classified sequences are summarized in the artificial group ‘Others’. Sites are designated by reclamation time (in years) (REC) or as undisturbed reference (UND).
(TIFF)

S1 Table **Relative abundances of bacterial phyla and proteobacterial classes in soils.** Values represent percentages of all sequences assigned to the domain Bacteria for all soils or individual soils.
(DOC)
