# Supplementary figures and images for: Succession of Bacterial Community Structure and Diversity in Soil along a Chronosequence of Reclamation and Re-Vegetation on Coal Mine Spoils in China
Source: PLoS One. 2014 Dec 11;9(12):e115024. doi: 10.1371/journal.pone.0115024 (PMC4263735; doi:10.1371/journal.pone.0115024)

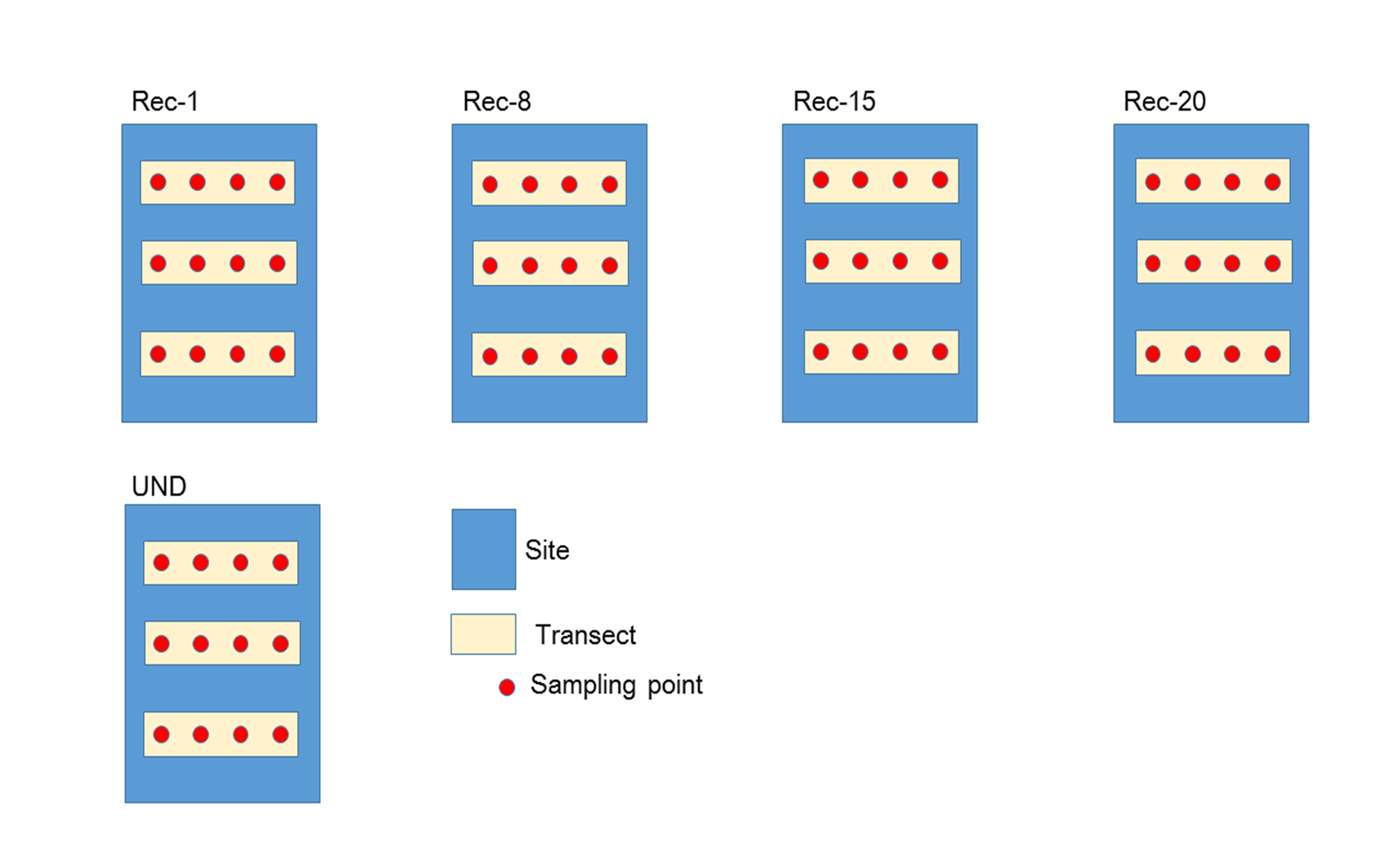

Supplement: S1 Figure — The schematic sampling map. UND refers to site undisturbed, REC-1 to site reclaimed for 1 year, REC-8 to site reclaimed for 8 years, REC-15 to site reclaimed for 15years and REC-20 to site reclaimed for 20 years. (TIF) [file pone.0115024.s001.tif]

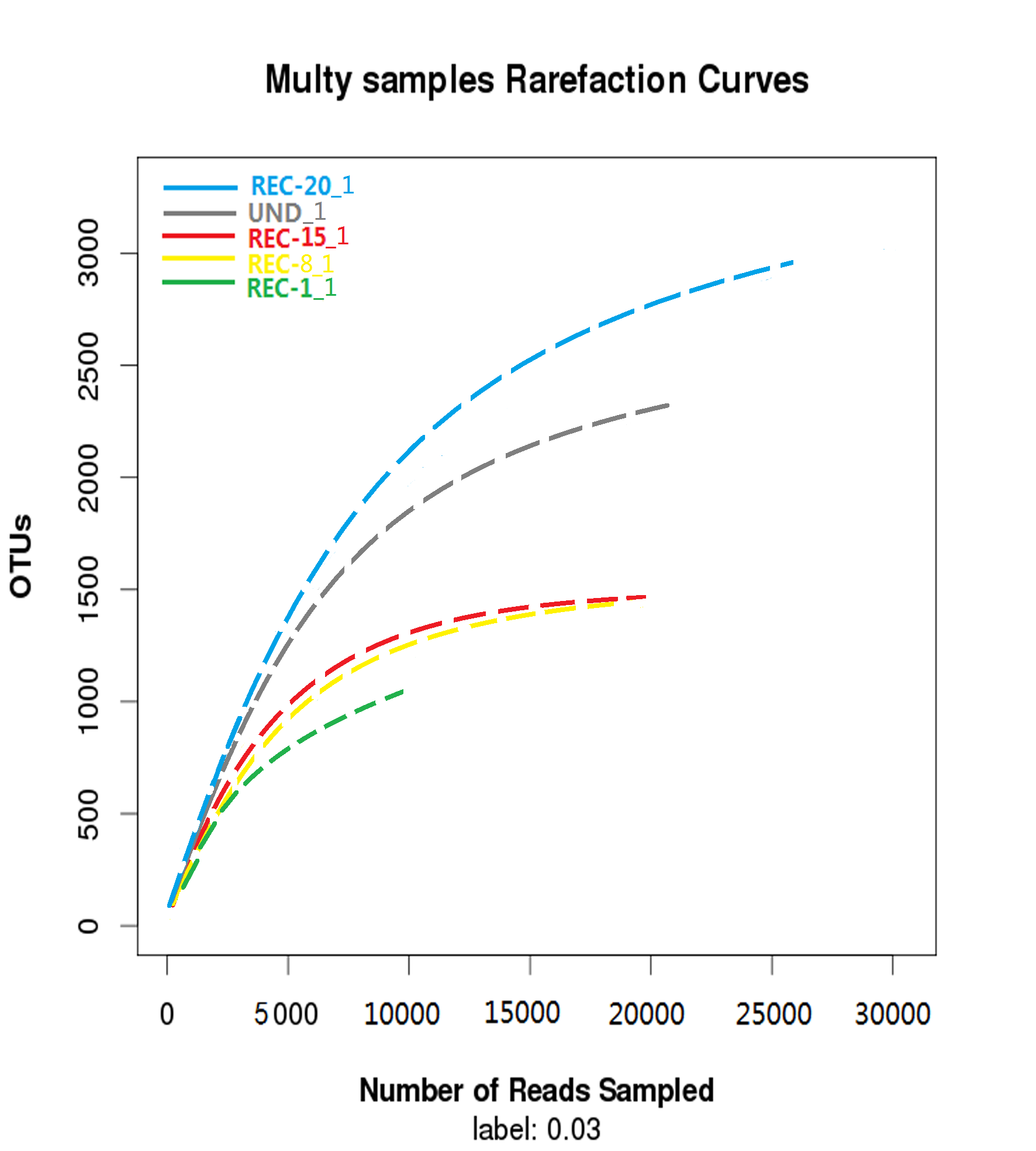

Supplement: S2 Figure — Rarefaction curves indicating the observed number of OTUs at a genetic distance of 3%. A, B and C represent three replicates for each site, respectively. Sites are designated by reclamation time (in years) (REC) or as undisturbed reference (UND). (ZIP) [file pone.0115024.s002.zip › Figure S2. Rarefaction curves/Figure S2(A).tif]

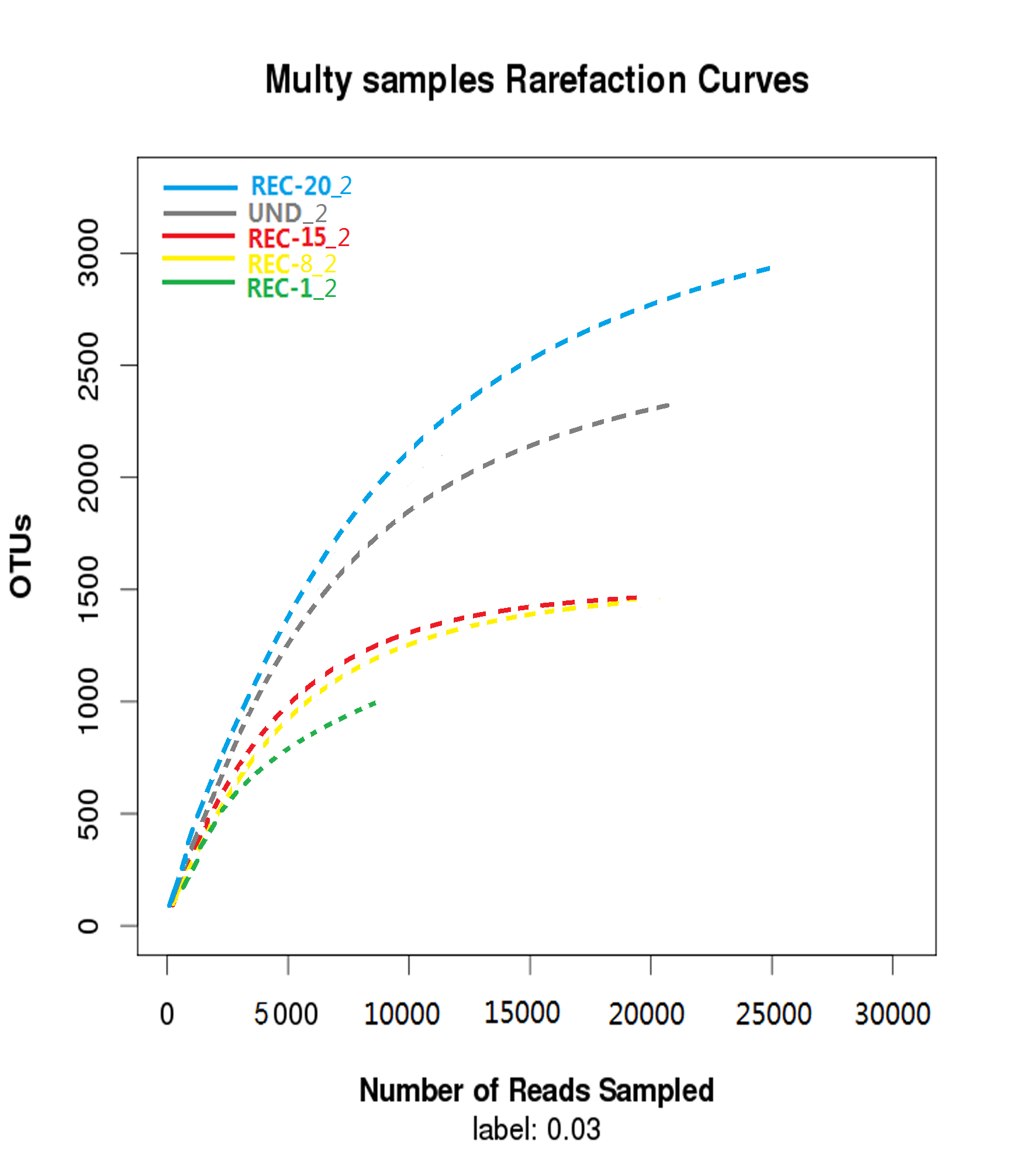

Supplement: S2 Figure — Rarefaction curves indicating the observed number of OTUs at a genetic distance of 3%. A, B and C represent three replicates for each site, respectively. Sites are designated by reclamation time (in years) (REC) or as undisturbed reference (UND). (ZIP) [file pone.0115024.s002.zip › Figure S2. Rarefaction curves/Figure S2(B).tif]

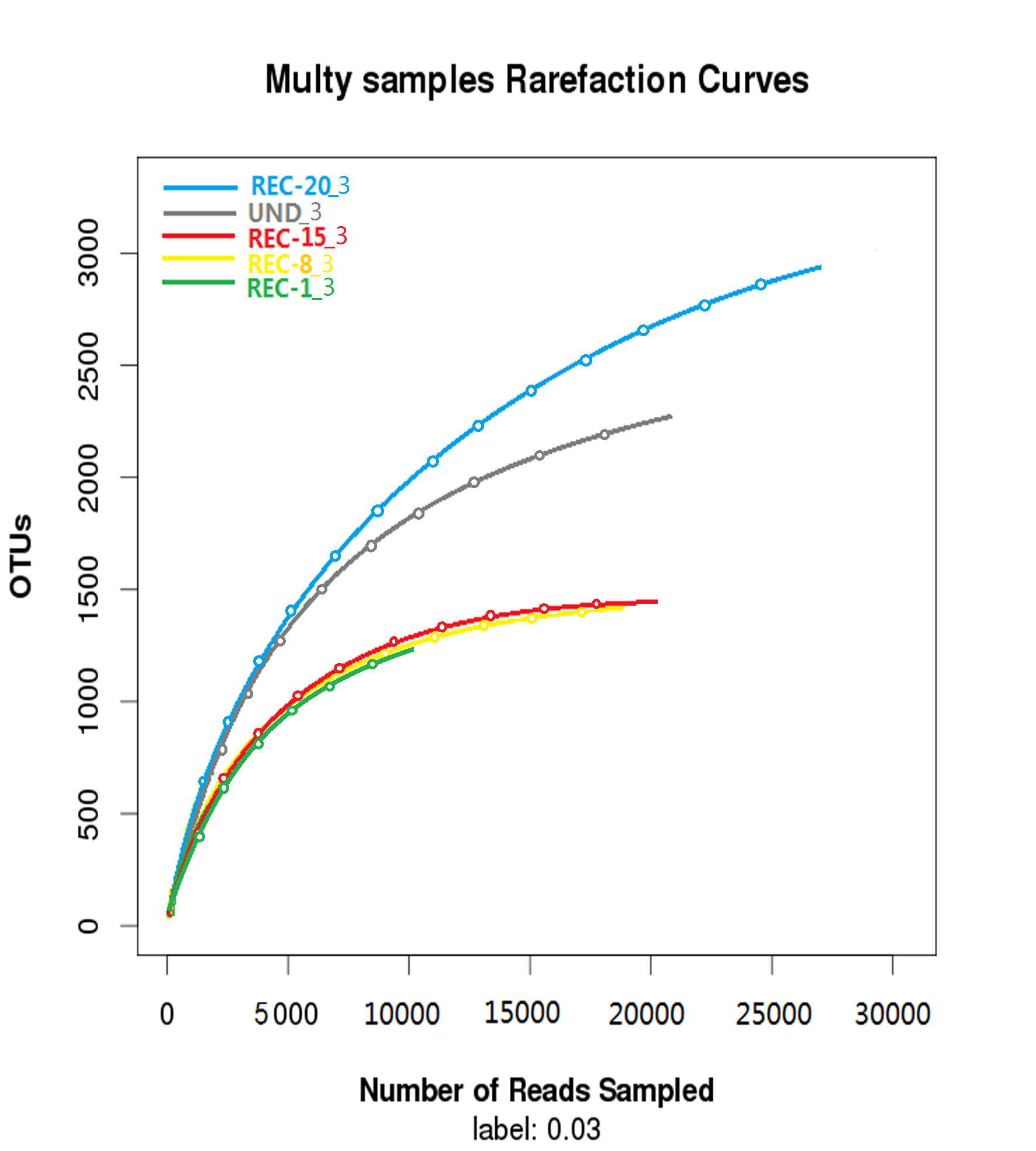

Supplement: S2 Figure — Rarefaction curves indicating the observed number of OTUs at a genetic distance of 3%. A, B and C represent three replicates for each site, respectively. Sites are designated by reclamation time (in years) (REC) or as undisturbed reference (UND). (ZIP) [file pone.0115024.s002.zip › Figure S2. Rarefaction curves/Figure S2(C).tif]

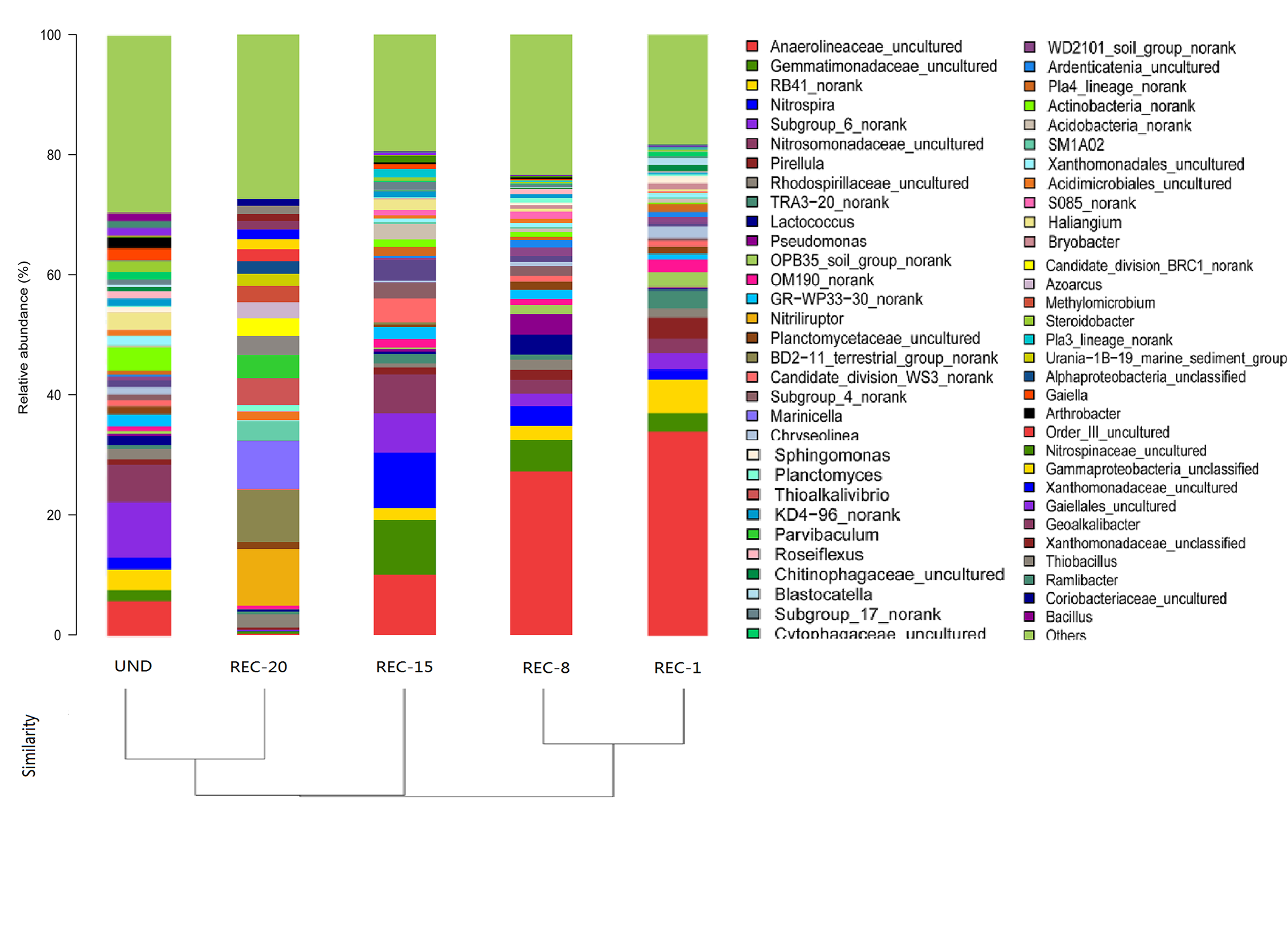

Supplement: S3 Figure — Microbial community barplot with cluster tree at the genus level. Phylogenetic groups accounting for ≤1% of all classified sequences are summarized in the artificial group ‘Others’. Sites are designated by reclamation time (in years) (REC) or as undisturbed reference (UND). (TIFF) [file pone.0115024.s003.tiff]
