# Supplementary material for: Succession of Bacterial Community Structure and Diversity in Soil along a Chronosequence of Reclamation and Re-Vegetation on Coal Mine Spoils in China
Source: PLoS One. 2014 Dec 11;9(12):e115024. doi: 10.1371/journal.pone.0115024 (PMC4263735; doi:10.1371/journal.pone.0115024)
Supplement: S1 Table — Relative abundances of bacterial phyla and proteobacterial classes in soils. Values represent percentages of all sequences assigned to the domain Bacteria for all soils or individual soils. (DOC) [file pone.0115024.s004.doc]

**Table S1.** **Relative abundances of bacterial phyla and proteobacterial classes in soils. Values represent percentages of all sequences assigned to the domain Bacteria for all soils or individual soils.**

| Phylogenetic group | Relative abundance (%) | | | | | |
| --- | --- | --- | --- | --- | --- | --- |
|  | Average | UND | REC-1 | REC-8 | REC-15 | REC-20 |
| *Alphaproteobacteria* | 10.79 | 16.17 | 7.60 | 3.35 | 10.78 | 16.04 |
| *Betaproteobacteria* | 6.27 | 4.22 | 7.63 | 8.86 | 5.47 | 5.18 |
| *Deltaproteobacteria* | 4.10 | 4.50 | 3.49 | 7.30 | 3.48 | 1.73 |
| *Gammaproteobacteria* | 12.95 | 16.73 | 5.21 | 5.06 | 13.47 | 24.28 |
| *Acidobacteria* | 7.82 | 10.25 | 0.20 | 5.58 | 9.58 | 13.51 |
| *Actinobacteria* | 9.20 | 4.42 | 17.52 | 14.01 | 7.08 | 2.99 |
| *Armatimonadetes* | 0.17 | 0.51 | 0.06 | 0.05 | 0.20 | 0.02 |
| *Bacteroidetes* | 4.80 | 3.27 | 3.32 | 7.16 | 5.45 | 4.81 |
| *Candidate_division_BRC1* | 0.00 | 0.00 | 0.02 | 0.00 | 0.00 | 0.00 |
| *Candidate_division_OD1* | 0.28 | 0.49 | 0.28 | 0.03 | 0.00 | 0.60 |
| *Candidate_division_OP11* | 0.23 | 0.14 | 0.19 | 0.00 | 0.80 | 0.00 |
| *Candidate_division_TM7* | 0.13 | 0.15 | 0.10 | 0.24 | 0.00 | 0.17 |
| *Candidate_division_WS3* | 0.79 | 0.97 | 0.01 | 0.00 | 1.02 | 1.93 |
| *Chlorobi* | 0.14 | 0.40 | 0.10 | 0.01 | 0.20 | 0.01 |
| *Chloroflexi* | 14.11 | 13.46 | 28.20 | 17.59 | 4.71 | 6.60 |
| *Cyanobacteria* | 0.05 | 0.02 | 0.04 | 0.00 | 0.16 | 0.04 |
| *Deferribacteres* | 0.01 | 0.00 | 0.00 | 0.00 | 0.03 | 0.00 |
| *Elusimicrobia* | 0.19 | 0.10 | 0.03 | 0.02 | 0.80 | 0.00 |
| *Fibrobacteres* | 0.30 | 0.13 | 0.06 | 0.00 | 1.31 | 0.00 |
| *Firmicutes* | 2.90 | 4.05 | 0.51 | 2.01 | 3.82 | 4.12 |
| *Fusobacteria* | 0.01 | 0.00 | 0.00 | 0.01 | 0.02 | 0.01 |
| *Gemmatimonadetes* | 4.97 | 5.42 | 0.44 | 3.45 | 7.49 | 8.07 |
| *Nitrospirae* | 2.28 | 3.50 | 0.13 | 0.95 | 5.17 | 1.66 |
| *No_Rank* | 8.41 | 0.00 | 14.88 | 15.30 | 10.86 | 1.01 |
| *Planctomycetes* | 7.31 | 9.27 | 8.90 | 7.50 | 5.83 | 5.07 |
| *Proteobacteria* | 34.11 | 41.62 | 23.94 | 24.57 | 33.20 | 47.23 |
| *Spirochaetae* | 0.02 | 0.02 | 0.00 | 0.00 | 0.02 | 0.04 |
| *TM6* | 0.04 | 0.03 | 0.03 | 0.04 | 0.00 | 0.10 |
| *Thermodesulfobacteria* | 0.01 | 0.00 | 0.00 | 0.00 | 0.03 | 0.00 |
| *Thermotogae* | 0.07 | 0.01 | 0.01 | 0.00 | 0.31 | 0.00 |
| *Verrucomicrobia* | 1.64 | 1.76 | 1.04 | 1.48 | 1.91 | 2.00 |
